# Physiological characterization of thermotolerant yeast for cellulosic ethanol production

**DOI:** 10.1007/s00253-014-5580-3

**Published:** 2014-02-18

**Authors:** Daniela A. Costa, Carlos J. A. de Souza, Patrícia S. Costa, Marina Q. R. B. Rodrigues, Ancély F. dos Santos, Mariana R. Lopes, Hugo L. A. Genier, Wendel B. Silveira, Luciano G. Fietto

**Affiliations:** 1 Departamento de Microbiologia (Department of Microbiology), Universidade Federal de Viçosa, Av. PH Rolfs s/n, Campus Universitário, 36571-000 Viçosa, MG Brazil; 2 Departamento de Bioquímica e Biologia Molecular (Department of Biochemistry and Molecular Biology), Universidade Federal de Viçosa, Av. PH Rolfs s/n, Campus Universitário, 36571-000 Viçosa, MG Brazil

**Keywords:** Simultaneous saccharification and fermentation, Ethanol, Sugarcane bagasse, Thermotolerant yeast

## Abstract

The conversion of lignocellulose into fermentable sugars is considered a promising alternative for increasing ethanol production. Higher fermentation yield has been achieved through the process of simultaneous saccharification and fermentation (SSF). In this study, a comparison was performed between the yeast species *Saccharomyces cerevisiae* and *Kluyveromyces marxianus* for their potential use in SSF process. Three strains of *S. cerevisiae* were evaluated: two are widely used in the Brazilian ethanol industry (CAT-1 and PE-2), and one has been isolated based on its capacity to grow and ferment at 42 °C (LBM-1). In addition, we used thermotolerant strains of *K. marxianus*. Two strains were obtained from biological collections, ATCC 8554 and CCT 4086, and one strain was isolated based on its fermentative capacity (UFV-3). SSF experiments revealed that *S. cerevisiae* industrial strains (CAT-1 and PE-2) have the potential to produce cellulosic ethanol once ethanol had presented yields similar to yields from thermotolerant strains. The industrial strains are more tolerant to ethanol and had already been adapted to industrial conditions. Moreover, the study shows that although the *K. marxianus* strains have fermentative capacities similar to strains of *S. cerevisiae,* they have low tolerance to ethanol. This characteristic is an important target for enhancing the performance of this yeast in ethanol production.

## Introduction

The production of first-generation ethanol occurs from the fermentation of sugars derived from agricultural raw materials, mainly corn and sugarcane. The USA and Brazil are responsible for 70 % of the world’s production of ethanol fuel (Argueso et al. 2009).

According to the Brazil’s National Company of Food and Supply (Companhia Nacional de Abastecimento (CONAB)), sugarcane production from the 2012/2013 harvest reached 602 million tonnes in Brazil, leading to the production of 39 million tonnes of sugar and 24 billion tonnes of ethanol. Because each tonne of sugarcane processed generates approximately 270–280 kg of bagasse, it can be extrapolated that 169 million tonnes of bagasse were produced (Canilha et al. 2012). Only one third of the biomass produced by the plant is used for sugar production, one third is bagasse, which is burnt to produce electricity, and the remaining one third is left in the field, which is decomposed by the microorganisms (Cortez et al. 2008). Thus, ethanol production would increase substantially if new technologies were developed to convert the polysaccharides from the leaves, straw, and bagasse, which represents approximately two thirds of the biomass, into fermentable sugars (Soccol et al. 2010).

The process of ethanol production from lignocellulosic wastes essentially consists of four stages: pretreatment, enzymatic hydrolysis, fermentation, and distillation (Sun and Cheng 2002; Tomas-Pejo et al. 2009). The traditional process of ethanol production from lignocellulosic biomass consists of a sequential process in which hydrolysis and fermentation are performed in different reactors. This process is known as separate hydrolysis and fermentation (SHF). An alternative to SHF is to perform hydrolysis and fermentation in the same bioreactor, a process known as simultaneous saccharification and fermentation (SSF) (Olofsson et al. 2008; Sanchez and Cardona 2008). The SSF process gives more attractive yields than the SHF process, with higher ethanol productivity and lower energy consumption. In SSF, the enzymes and microorganisms are added to the same reactor, allowing the monosaccharides released during enzymatic hydrolysis of biomass to be immediately converted into ethanol, and the continuous removal of monosaccharides from the medium minimizes the inhibition of enzymes by their products (Ballesteros et al. 2004; Olsson et al. 2006; Sanchez and Cardona 2008).

However, one of the major drawbacks of the SSF process is that enzymatic hydrolysis has an optimum temperature of approximately 50 °C, while most fermentative microorganisms have optimum temperatures ranging between 30 and 37 °C (Abdel-Banat et al. 2010; Krishna et al. 2001). In this regard, an alternative to achieving the SSF process is to use thermotolerant fermentative microorganisms (Suryawati et al. 2008). There are several advantages associated with using thermotolerant yeasts, such as cost reduction associated with cooling fermentation vats, obtaining higher yields in saccharification, continuous removal of ethanol, and decreased risk of bacterial contamination (Banat et al. 1998; Limtong et al. 2007).

Strains of *Saccharomyces cerevisiae*, a traditional yeast for alcoholic fermentation, are widely used in the ethanol fuel industry. In Brazil, the strains most used by the Brazilian industries are PE-2, CAT-1 and BG-1 because they have remarkable capacities to compete with native strains and to survive and dominate during the industrial fermentation process. In the 2007/2008 crop period, the PE-2 and CAT-1 strains were used in approximately 150 distilleries, representing approximately 60 % of the ethanol fuel produced in Brazil (Basso et al. 2008). Most *S. cerevisiae* strains are tolerant to low pH values, high concentrations of sugars, and ethanol compared to other species, which contributes to reducing the risk of contamination during industrial fermentation (Nevoigt 2008). However, the fermentation efficiency of *S. cerevisiae* at temperatures above 35 °C is low (Ohta et al. 1988). Accordingly, *S. cerevisiae* strains capable of fermenting at temperatures above 35 °C have been isolated, mainly from tropical regions (Banat et al. 1998; De Souza et al. 2012).

Among the yeasts used in industrial processes, *Kluyveromyces marxianus* has the best performance for growth and fermentation at temperatures above 35 °C (Abdel-Banat et al. 2010; Abdel-Fattah et al. 2000; Ballesteros et al. 2004; Banat et al. 1998; Hong et al. 2007; Nonklang et al. 2008; Singh et al. 1998; Suryawati et al. 2008; Wilkins et al. 2008). In addition, *K. marxianus* has other characteristics that are desirable in fermentation processes, such as high growth rates and the capacity to utilize a wide variety of substrates (Fonseca et al. 2008). In this context, this yeast species has been reported to be the most promising for use in SSF processes (Suryawati et al. 2008).

Recently, (De Souza et al. (2012)) have demonstrated the potential of the *S. cerevisiae* LBM1 strain to produce ethanol from sugarcane bagasse by the SSF process.

Considering that the tolerance of yeast to high temperatures and high concentrations of sugars and ethanol are desirable characteristics in industrial applications (Banat et al. 1992), the influence of these factors on the growth of six strains of yeasts was evaluated in this study: three strains of *S. cerevisiae*, two of which are widely used in the Brazilian ethanol industry (CAT-1 and PE-2) (Basso et al. 2008) and one strain isolated based on its capacity to grow and ferment at 42 °C (LBM-1) (De Souza et al. 2012); and three strains of *K. marxianus*, of which two were obtained from biological collections, ATCC 8554 and CCT 4086, and one strain was isolated because of its fermentative capacity (UFV-3) (De Souza et al. 2012; Diniz et al. 2012; Dos Santos et al. 2013; Silveira et al. 2005). In addition, the performances of these six strains of yeast in the production of ethanol from sugarcane bagasse in an SSF process were compared.

## Materials and methods

### Strains and culture media

The yeast strains used in this study are listed in Table 1. The culture medium used for maintenance and activation was YPD [2 % yeast extract, 1 % peptone, and 2 % glucose (*w*/*v*)]; for solid medium, 2 % (*w*/*v*) agar was added. All strains were stored and maintained in 20 % glycerol at −80 °C.

**Table 1:** Strains of yeasts used

Yeast strain	Genotype	Source	Strain designations
*Saccharomyces cerevisiae* LBM-1	Wild type	Laboratório Biotecnologia Molecular-DBB/UFV	UFMG-CM-Y342-Centro de Coleções Taxonômicas da UFMG
*Saccharomyces cerevisiae* CAT-1	Industrial strain	Fermentec-Piracicaba - Brazil	CBMAI 0957-Coleção Brasileira de Micro-organismos de Ambiente e Indústria
*Saccharomyces cerevisiae* PE-2	Industrial strain	Fermentec-Piracicaba-Brazil	CBMAI 0959-Coleção Brasileira de Micro-organismos de Ambiente e Indústria
*Kluyveromyces marxianus* UFV-3	Wild type	Laboratório de Fisiologia de Micro-organismos UFV	_
*Kluyveromyces marxianus* ATCC 8554	Wild type	American Type Culture Collection	CBS 5795 = NRRL Y-1109 = ATCC 8554
*Kluyveromyces marxianus* CCT 4086	Wild type	Coleção de Culturas Tropicais-André Tosello Tropical Culture	CBS 397 = NRRL Y-2415 = ATCC 46537

### Culturing conditions in solid media

Strains of *S. cerevisiae* (LBM-1, CAT-1, and PE-2) and *K. marxianus* (UFV-3, ATCC 8554 and CCT 4086) were previously grown in 2 % YPD medium with agitation at 180 rpm at 28 °C for testing tolerance to temperature, ethanol, and glucose concentrations. Serial dilutions (1:1, 1:10, and 1:100) were prepared in saline solution (0.85 % NaCl) from cultures with optical densities (O.D._600 nm_) of 0.5. A volume of 5 μl of each dilution of culture was applied onto solid YPD plates. The tests were conducted on plates with different concentrations of carbon source (2, 4, 8, and 16 % glucose) and in the presence or absence of ethanol (2, 4, 6, and 8 % ethanol in 4 % YPD). Plates were sealed with Parafilm and were incubated at 28, 37, 42, and 45 °C for 72 h.

### Culturing conditions in liquid media

Tests of tolerance to temperature, ethanol, and glucose concentrations were performed in 96-well microplates incubated under agitation in a Versamax apparatus (Molecular Devices microplate reader). Strains of *S. cerevisiae* (LBM-1, CAT-1, and PE-2) and *K. marxianus* (UFV-3, ATCC 8554, and CCT 4086) were previously grown in 2 % YPD medium under agitation at 180 rpm at 28 °C. From the preinoculums, dilutions were made in saline solution to an initial O.D._600 nm_ of 0.1. Each strain was inoculated in a final volume of 200 μl of medium, and each test was performed in triplicate. Tests for growth were performed at different glucose concentrations (2, 4, 8, and 16 %) and in the presence or absence of ethanol (ethanol 2, 4, 6, and 8 % in 4 % YPD medium). The plates were sealed using plastic (Axygen Platemax Axysel sealing film) and incubated in the reader at temperatures of 28, 37, 42, and 45 °C for 16 h. Data were collected using the SoftMax Pro 5.3 program installed on the Versamax reader.

The specific growth rate of culture on each substrate was determined by linear regression of the values obtained by the Napierian logarithm of the O.D._600 nm_ during the exponential growth phase, using at least five points of a plot of O.D._600 nm_ versus time. The specific growth rate (*μ*) is the slope of the line obtained by regression.

### Fermentation tests

Fermentation tests were performed at 37 and 42 ºC using glucose and pretreated sugarcane bagasse as substrates. Yeasts were preactivated in 50-mL Erlenmeyer flasks containing 20 mL minimal media: 50 mM citrate buffer, pH 4.8; yeast extract (2.5 g L^−1^); peptone (2.5 g L^−1^); NH_4_Cl (2 g L^−1^); KH_2_PO4 (1 g L^−1^); MgSO_4_.7H_2_O (0.3 g L^−1^) with added glucose (20 g L^−1^) with agitation at 180 rpm for 16 h at 28 °C. Fermentation tests were then conducted in 125-mL Erlenmeyer flasks containing 50 mL of minimal medium supplemented with 4 % (*w*/*v*) glucose. Cell cultures were inoculated with an initial O.D._600 nm_ of 2, and the test was conducted at 37 and 42 °C for 12 h at 180 rpm.

SSF tests were conducted in accordance with the parameters established by (De Souza et al. 2012). SSF experiments were conducted in 125-mL Erlenmeyer flasks each containing 50 mL of minimal medium without glucose, as described above, with agitation at 180 rpm. The solid fraction obtained from pretreatment was used as the substrate at 8 % (*w*/*v*) concentration. Initially, a presaccharification was made for 72 h at 50 °C using commercial enzyme (Celluclast 1.5 L) at a concentration of 15 filter paper units (FPU) per gram of substrate. SSF tests were performed under sterile conditions, and fermentation was conducted at 37 and 42 °C using yeasts at an initial O.D._600 nm_ of 2. In both tests, fermentation samples were withdrawn at 2-h intervals for subsequent determination of glucose and ethanol by high-performance liquid chromatography (HPLC). SSF tests were also conducted in bioreactor. By this purpose, the experiments were performed in a 1.3 L bioreactor (New Brunswick) containing 500 mL of minimal medium without glucose as described above, and 40 g of pretreated sugar cane bagasse. Initially, a prehydrolysis was carried out for 72 h at 50 °C using a commercial enzyme (Celluclast 1.5 L) 15 FPU per gram of substrate. After this initial period, the fermentation was initiated using the strain of *S. cerevisiae* CAT-1. This step was conducted at 37 °C and aliquots were collected for analysis of glucose and ethanol by HPLC.

### Fermentation parameters

The ethanol yield (*Y*_E/B_) was calculated at the end (8 h) of fermentation by dividing the difference between the final (EtOH_f_) and initial (EtOH_i_) ethanol masses (in grams) by the initial pretreated biomass (in grams):

$$ {Y}_{\mathrm{E} / \mathrm{B}}=\frac{{\mathrm{E}\mathrm{tOH}}_{\mathrm{f}}-{\mathrm{E}\mathrm{tOH}}_{\mathrm{i}}}{\mathrm{Biomass}} $$

The ethanol yield (*Y*_E/G_) was calculated at the end (8 h) of fermentation by dividing the difference between the final (EtOH_f_) and initial (EtOH_i_) ethanol masses (in grams) by difference between the final (Glu_f_) and initial (Glu_i_) glucose masses:

$$ {Y}_{\mathrm{E} / \mathrm{G}}=\frac{{\mathrm{E}\mathrm{tOH}}_{\mathrm{f}}-{\mathrm{E}\mathrm{tOH}}_{\mathrm{i}}}{{\mathrm{Glu}}_{\mathrm{f}}-{\mathrm{Glu}}_{\mathrm{i}}} $$

### Analytical methods

Cell density was measured by turbidity at 600 nm. To determine the concentrations of ethanol and sugar in the culture media, samples were collected and subjected to centrifugation. The supernatant was kept at −20 °C for analysis. Quantitative analysis of sugars and ethanol were conducted by HPLC using an Aminex HPX-87H (Bio-Rad) ion exchange column maintained at 60 °C. The eluent for the separation was 5 mM sulfuric acid, applied at an elution rate of 0.7 mL min^−1^. The column was attached to an HP 1047 A refractive index detector.

## Results

### Temperature and glucose concentrations effects on the growth of *K. marxianus* and *S. cerevisiae* strains

To analyze the temperature and the concentrations of sugars and ethanol effects on the growth of yeasts, initial tests were performed on solid media at different glucose concentrations (2, 4, 8, and 16 % *w*/*v*) under different temperatures (30, 37, 42, and 45 °C). As shown in Table 2, the tested strains grew in all dilutions and in all concentrations of glucose up to 42 °C. At 45 °C, the *K. marxianus* strains grew in the four glucose concentrations tested, while the *S. cerevisiae* strains were able to grow only in media containing glucose concentrations of 8 and 16 % (*w*/*v*) (Table 2).

**Table 2:** Growth evaluation of yeast strains in YP medium containing different concentrations of glucose (2, 4, 8, and 16 % *w*/*v*) and at different temperatures, with (++++) good growth, (++) average growth, (+) weak growth, (−) no growth

Yeast strain	30 °C				37 °C				42 °C				45 °C			
	2 %	4 %	8 %	16 %	2 %	4 %	8 %	16 %	2 %	4 %	8 %	16 %	2 %	4 %	8 %	16 %
*S. cerevisiae* LBM-1	+++	+++	+++	+++	+++	+++	+++	+++	+++	+++	+++	+++	−	+	+++	+++
*S. cerevisiae* CAT-1	+++	+++	+++	+++	+++	+++	+++	+++	+++	+++	+++	+++	−	−	++	+++
*S. cerevisiae* PE-2	+++	+++	+++	+++	+++	+++	+++	+++	+++	+++	+++	+++	−	−	++	+++
*K. marxianus* UFV-3	+++	+++	+++	+++	+++	+++	+++	+++	+++	+++	+++	+++	+++	+++	+++	+++
*K. marxianus* ATCC 8554	+++	+++	+++	+++	+++	+++	+++	+++	+++	+++	+++	+++	+++	+++	+++	+++
*K. marxianus* CCT 4086	+++	+++	+++	+++	+++	+++	+++	+++	+++	+++	+++	+++	+++	+++	+++	+++

The inhibitory effect of ethanol on yeast growth was evaluated at different temperatures. As illustrated in Table 3, the stress caused by ethanol in yeasts was more drastic than the one caused by variations in glucose concentration. At 30 °C, *S. cerevisiae* strains exhibited good growth in up to 8 % (v/v) ethanol and weak growth at 10 % (v/v) ethanol, while the *K. marxianus* strains only grew up to a concentration of 6 % (v/v) ethanol. However, the tolerance of the *S. cerevisiae* strains to ethanol decreased with increasing temperature. At 30 °C, ethanol tolerance was 10 % (v/v), and it decreased to 4 % (v/v) at 42 °C. For *K. marxianus*, the increase in temperature also reduced ethanol tolerance, but the effect was less drastic than for *S. cerevisiae*. At 30 °C, the ethanol tolerances for *S. cerevisiae* and *K. marxianus* strains were 10 % and 6 % (v/v), respectively, while at 42 °C, the tolerance was 4 % (v/v) for both.

**Table 3:** Growth evaluation of yeast strains in 4 % YPD medium containing different concentrations of ethanol (2, 4, 6, 8, and 10 % v/v) and at different temperatures, with (++++) good growth, (++) average growth, (+) weak growth, (−) no growth

Yeast strain	30 °C					37 °C					42 °C					45 °C				
	2 %	4 %	6 %	8 %	10 %	2 %	4 %	6 %	8 %	10 %	2 %	4 %	6 %	8 %	10 %	2 %	4 %	6 %	8 %	10 %
*S. cerevisiae* LBM-1	+++	+++	+++	+++	+	+++	+++	+++	++	−	+++	+++	−	−	−	+++	−	−	−	−
*S. cerevisiae* CAT-1	+++	+++	+++	+++	+	+++	+++	+++	+++	−	+++	+++	−	−	−	+++	−	−	−	−
*S. cerevisiae* PE-2	+++	+++	+++	+++	+	+++	+++	+++	++	−	+++	+++	−	−	−	+++	−	−	−	−
*K. marxianus* UFV-3	+++	+++	+++	−	−	+++	+++	−	−	−	+++	+	−	−	−	+++	−	−	−	−
*K. marxianus* ATCC 8554	+++	+++	+++	+	−	+++	+++	−	−	−	+++	+++	−	−	−	+++	+++	−	−	−
*K. marxianus* CCT 4086	+++	+++	+++	−	−	+++	+++	−	−	−	+++	+++	−	−	−	+++	+++	−	−	−

**Fig. 1:**
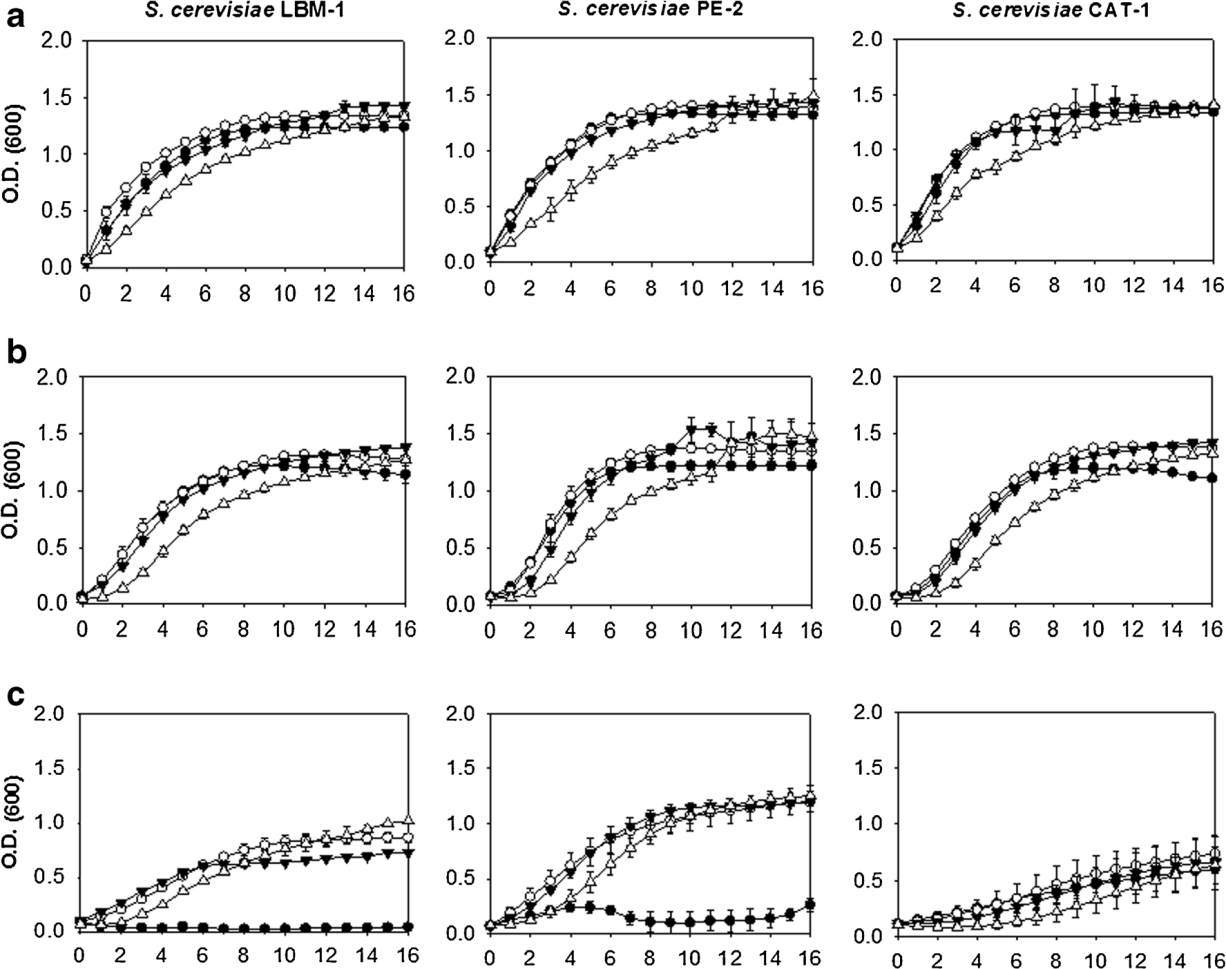
Growth curves of *S. cerevisiae* strains as a function of varying temperature and glucose concentrations in the liquid YP medium. Each *column* represents a strain, LBM-1, PE-2, and CAT-1, as shown in the figure. Each *line* represents a specific growth temperature. **a** 30 °C. **b** 37 °C. **c** 42 °C. The *symbols* represent the concentration of glucose added to the liquid culture medium: (*black circle*) 2 % glucose, (*white circle*) 4 % glucose, (*black down-pointing triangle*) 8 % glucose and (*white triangle*) 16 % glucose

To evaluate the growth of yeast in liquid medium, yeasts were exposed to thermal, osmotic, and ethanol stresses, and their growth was monitored for 16 h. Figure 1 represents the growth of *S. cerevisiae* strains at different concentrations of glucose at different temperatures.

**Fig. 2:**
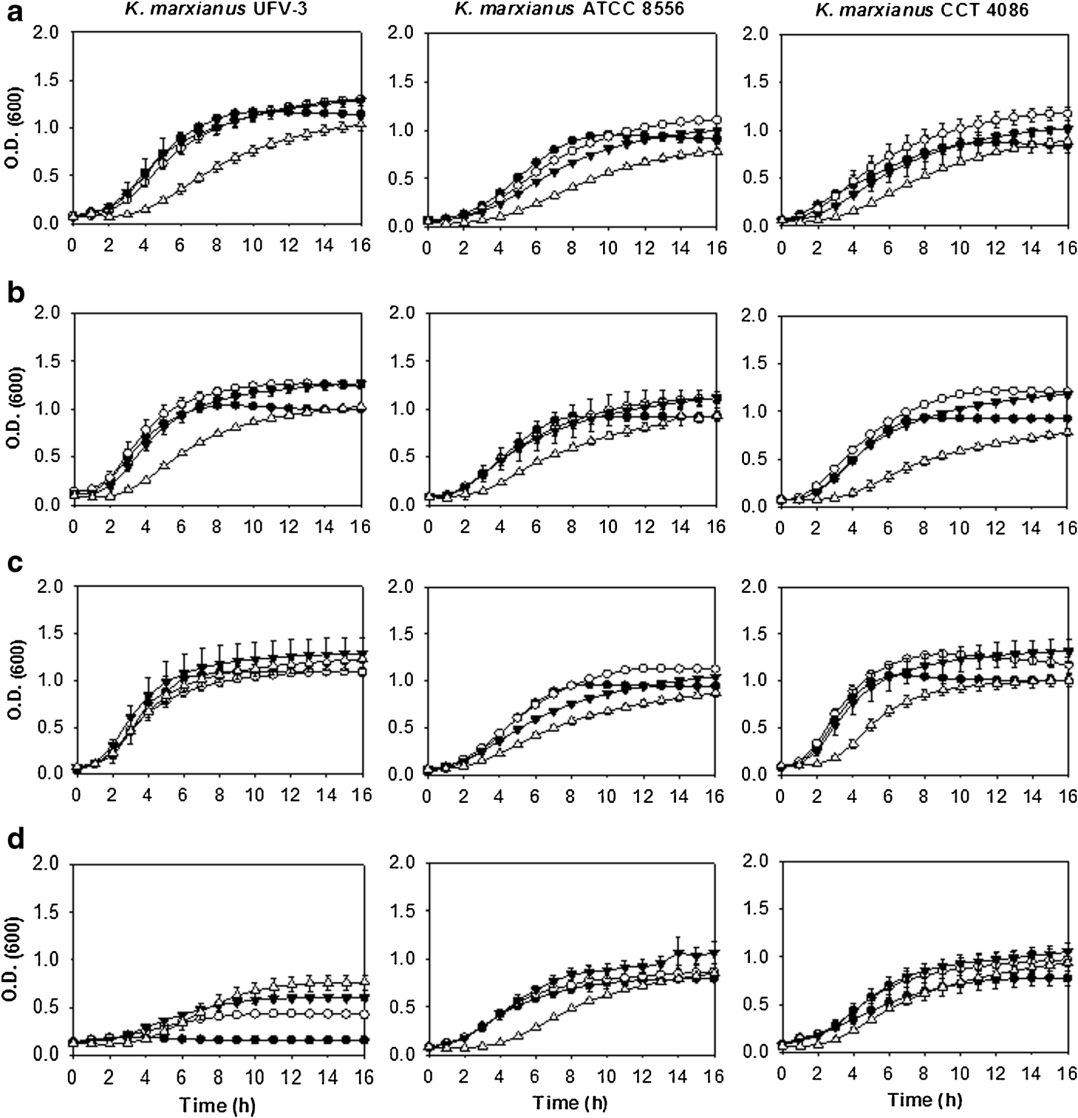
Growth curves of *K. marxianus* strains as a function of varying temperature and glucose concentrations in the liquid YP medium. Each *column* represents a strain, UFV-3, ATCC 8554, and CCT 4086, as shown in the figure. Each *line* represents a specific growth temperature. **a** 30 °C, **b**. 37 °C. **c** 42 °C. **d** 45 °C. The *symbols* represent the concentration of glucose added to the liquid culture medium: (*black circle*) 2 % glucose, (*white circle*) 4 % glucose, (*black down-pointing triangle*) 8 % glucose, and (*white triangle*) 16 % glucose

When evaluating the effects of glucose concentration on growth, it was noted that *S. cerevisiae* strains were not dramatically affected by increased glucose concentrations, but *K. marxianus* strains showed less growth in 16 % glucose (*w*/*v*) (Fig. 2b) compared to *S. cerevisiae* strains (Fig. 1b). The high glucose concentrations effects was higher for *K. marxianus* CCT 4086, for which the lag phase increased; additionally, a maximum O.D._600 nm_ of 0.78 ± 0.038 was attained, compared with O.D._600 nm_ values of 1.0 to 1.5 for the other strains (Table 4).

**Table 4:** Specific growth rates (*μ*) and maximum O.D._600 nm_ values in microplates containing 4 % YPD medium at different temperatures

Yeast strain	*μ* value (h^−1^)				O.D._(600)_			
	30 °C	37 °C	42 °C	45 °C	30 °C	37 °C	42 °C	45 °C
LBM-1	0.13	0.22	0.27	0.11	1.18	1.08	0.61	0.15
CAT-1	0.18	0.37	0.23	0.03	1.28	1.24	0.86	0.15
PE-2	0.15	0.32	0.20	0.06	1.27	1.10	0.34	0.13
UFV-3	0.46	0.40	0.38	0.17	0.79	1.05	0.87	0.34
ATCC 8554	0.41	0.39	0.38	0.32	0.56	0.72	0.75	0.63
CCT 4086	0.36	0.35	0.38	0.33	0.73	0.89	1.17	0.68

Regarding temperature, at 42 °C (Fig. 2c), *K. marxianus* strains grew better in relation to the *S. cerevisiae* strains (Fig. 1c), confirming the already described thermotolerance of *K. marxianus. S. cerevisiae* strains LBM-1 and CAT-1 did not grow at 42 °C in 2 % (*w*/*v*) glucose (Fig. 1c); growth only occurred in glucose concentrations above 2 % (*w*/*v*). *S. cerevisiae* PE-2 grew less compared to the LBM-1 and CAT-1 strains under the same conditions.

At 45 °C (Fig. 2d), *K. marxianus* strains grew at different concentrations of glucose, while *S. cerevisiae* strains did not grow in the glucose concentrations tested. For *K. marxianus* UFV-3, the same phenomenon was observed for *S. cerevisiae* LBM-1 and *S. cerevisiae* CAT-1, i.e., higher glucose concentrations seemed to protect the cells from stress caused by increased temperature.

To verify the strains’ thermotolerance capacities, a comparison of the specific growth rates (*μ*) (h^−1^) was made at different temperatures. For calculation of the *μ* values, a glucose concentration of 4 % (*w*/*v*) was chosen, as the strains showed better growth at different temperatures at that concentration.

As noted in Table 4, the specific growth rates of *S. cerevisiae* strains at 30 °C were similar. At a temperature of 37 °C, *S. cerevisiae* CAT-1 and *S. cerevisiae* PE-2 had the greatest μ values compared to LBM-1: 0.368 and 0.318, respectively. At 42 °C, the three *S. cerevisiae* strains grew similarly. At 45 °C, the growth of *S. cerevisiae* strains decreased substantially; the only strain that still showed growth was *S. cerevisiae* LBM-1. With these data, it can be concluded that the maximum tolerated temperature under the conditions tested for *S. cerevisiae* strains was 42 °C. Another fact to be noted was the more thermotolerant behavior of the *S. cerevisiae* LBM-1 strain.

The growth rates of *K. marxianus* were much higher than those of *S. cerevisiae* (Table 4). *K. marxianus* UFV-3 had similar μ values for temperatures of 30, 37, and 42 °C, while the growth rate decreased at 45 °C. However, the biological collection *K. marxianus* strains ATCC 8554 and CCT 4086 had similar *μ* values. These thermotolerance data are consistent with the literature because it has already been demonstrated that *K. marxianus* grows rapidly, even at temperatures above 40 °C (Fonseca et al. 2008).

### Ethanol effects on the growth of *K. marxianus* and *S. cerevisiae* strains

**Fig. 3:**
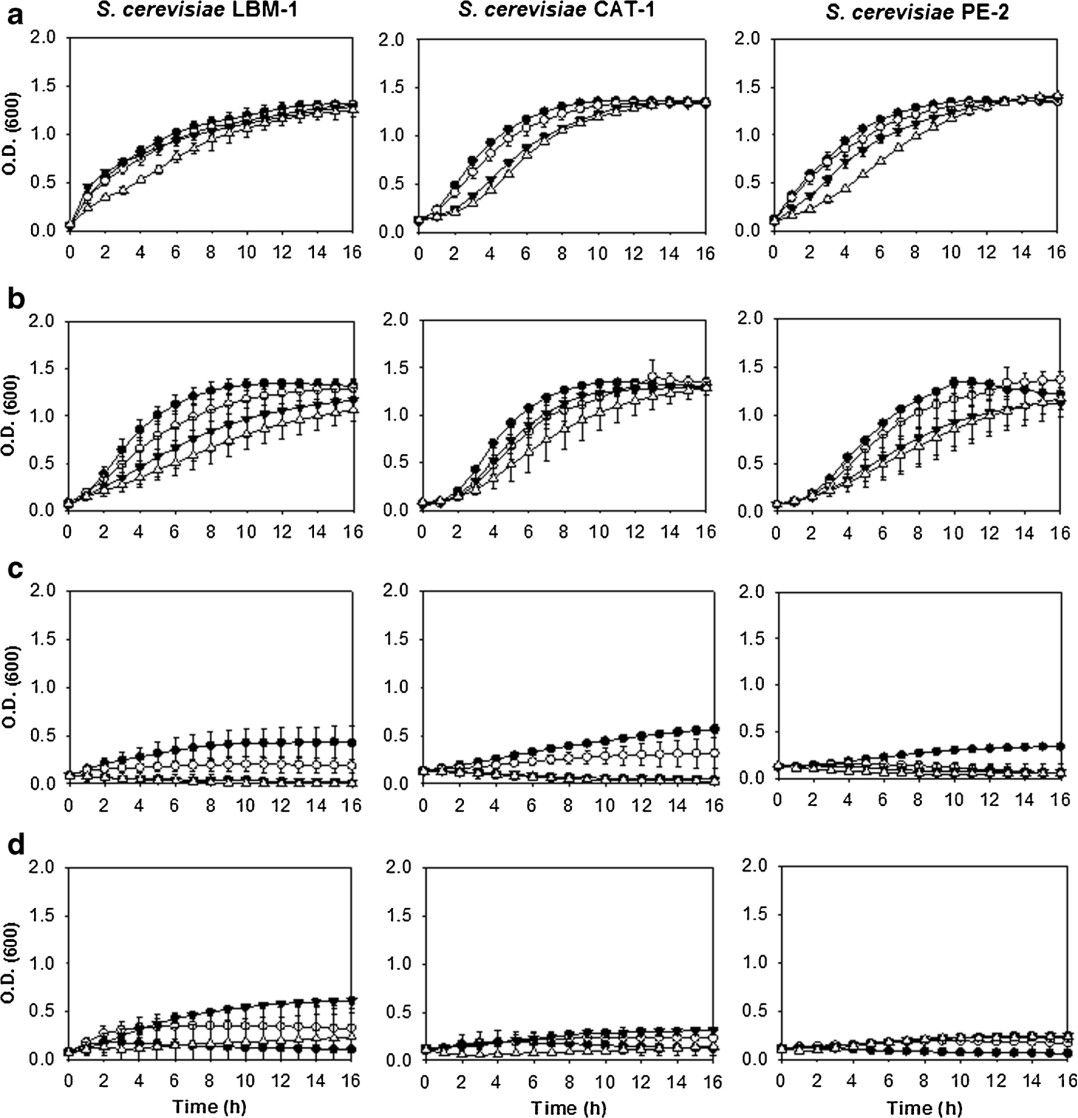
Growth curves of *S. cerevisiae* strains as a function of varying temperature and ethanol concentrations in the liquid YP medium supplemented with 4 % glucose (v/v). Each *column* represents a strain, LBM-1, PE-2, and CAT-1, as shown in the figure. Each *line* represents a specific growth temperature. **a** 30 °C. **b** 37 °C. **c** 42 °C. **d** 45 °C. The *symbols* represent the concentration of ethanol added to the liquid culture medium: (*black circle*) 2 % ethanol, (*white circle*) 4 % ethanol, (*black down-pointing triangle*) 6 % ethanol, and (*white triangle*) 8 % ethanol

**Fig. 4:**
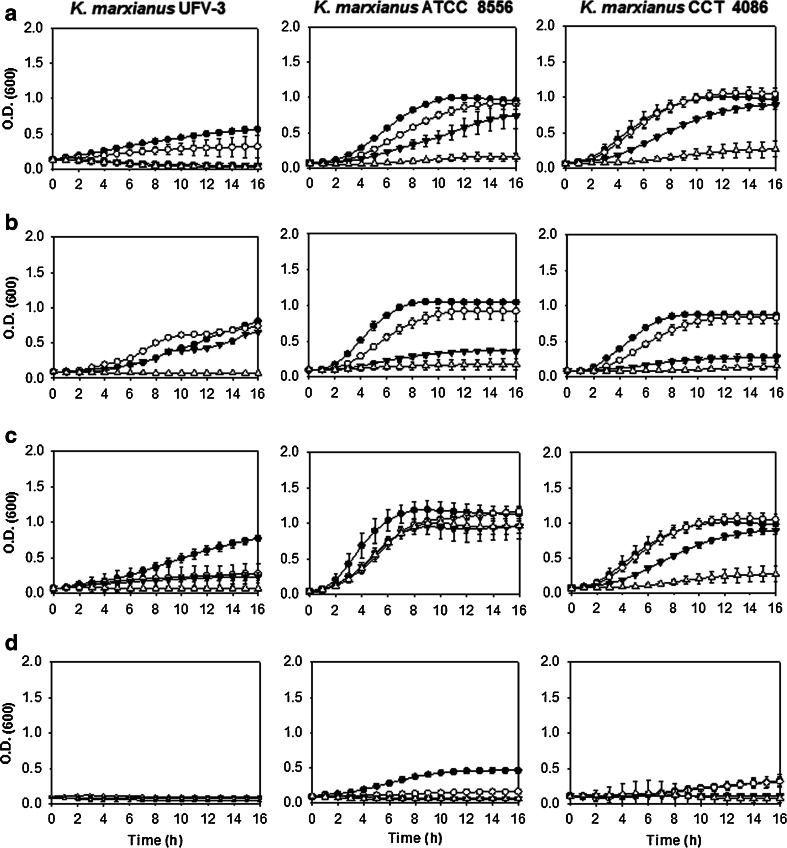
Growth curves of *K. marxianus* strains as a function of varying temperature and ethanol concentrations in the liquid YP medium supplemented with 4 % glucose (v/v). Each column represents a strain, UFV-3, ATCC 8554, and CCT 4086, as shown in the figure. Each *line* represents a specific growth temperature. **a** 30 °C. **b** 37 °C. **c** 42 °C. **d** 45 °C. The *symbols* represent the concentration of ethanol added to the liquid culture medium: (*black circle*) 2 % ethanol, (*white circle*) 4 % ethanol, (*black down-pointing triangle*) 6 % ethanol, and (*white triangle*) 8 % ethanol

Figures 3 and 4 represent the growth variations of the strains tested in different concentrations of ethanol: 2, 4, 6, and 8 % (v/v). *S. cerevisiae* strains tolerated ethanol to concentrations up to 8 % (v/v), and the newly isolated *S. cerevisiae* LBM-1 strain proved to be as tolerant to ethanol as the industrial strains *S. cerevisiae* CAT-1 and *S. cerevisiae* PE-2 (Fig. 3). However, *K. marxianus* had lower tolerances to ethanol: the *K. marxianus* biological collection strains ATCC 8554 and CCT 4086 tolerated higher concentrations of ethanol, up to 6 % (v/v), than the *K. marxianus* UFV-3 strain, which grew only in concentrations up to 4 % (v/v) ethanol (Fig. 4).

In Fig. 4b, it is shown that increasing the temperature to 37 °C did not significantly change the growth of *S. cerevisiae* strains; however, when growth was assessed at the higher temperatures of 42 °C (Fig. 4c) and 45 °C (Fig. 4d), *S. cerevisiae* growth was reduced, and at 42 °C, no strain was able to grow in the higher ethanol concentrations of 6 and 8 % (v/v). Therefore, tests at 45 °C were not performed. The *K. marxianus* strains were less tolerant to ethanol compared with the *S. cerevisiae* strains.

### Fermentation tests

The thermotolerance observed in the strains tested is of utmost importance in making the SSF process from sugarcane bagasse viable. Thus, two temperatures were selected—37 and 42 °C, at which all of the tested strains were able to grow—to perform fermentation and SSF tests.

The fermentation potential of *S. cerevisiae* and *K. marxianus* was evaluated in fermentation medium with 4 % (*w*/*v*) added glucose for 12 h at 37 and 42 ºC (Table 5). At 37 °C, *S. cerevisiae* and *K. marxianus* strains consumed all of the glucose present in the medium, producing 20 g L^−1^ of ethanol on average, resulting in similar fermentation yields for the strains at this temperature.

**Table 5:** Fermentation at 37 and 42 °C with an initial O.D._600 nm_ of 2 in 50 mL of fermentation medium supplemented with 4 % (*w*/*v*) glucose

Yeast strain	Initial glucose (g L^−1^)		Final glucose (g L^−1^)		Ethanol (g L^−1^)		Y_E/G_	
	37 °C	42 °C	37 °C	42 °C	37 °C	42 °C	37 °C	42 °C
LBM-1	47.00	47.00	0.04	10.35	21.70	10.64	0.46	0.29
CAT-1	47.00	47.00	0.00	9.82	22.77	9.92	0.48	0.27
PE-2	47.00	47.00	1.20	17.19	22.24	8.47	0.49	0.28
UFV-3	47.00	47.00	1.93	16.11	18.53	7.24	0.41	0.23
ATCC 8554	47.00	47.00	0.00	0.33	19.66	12.11	0.42	0.26
CCT 4086	47.00	47.00	0.00	0.05	18.10	12.06	0.39	0.26

At 42 °C, the strains showed similar behavior; however, residual glucose was higher for *S. cerevisiae* LBM-1, *S. cerevisiae* CAT-1, *S. cerevisiae* PE-2, and *K. marxianus* UFV-3, while *K. marxianus* ATCC 8554 and *K. marxianus* CCT 4086 consumed all of the glucose in the medium. The final production of ethanol was very similar for all strains, with higher values for *K. marxianus* ATCC 8554 and *K. marxianus* CCT 4086: 12.11 g L^−1^ and 12.06 g L^−1^ of ethanol, respectively (Table 5). The yield of ethanol at 42 °C was slightly higher for the *S. cerevisiae* yeast species than for the *K. marxianus* yeasts.

### Simultaneous saccharification and fermentation test with sugarcane bagasse

Table 6 describes the concentrations and yields obtained by the SSF process at different temperatures for the strains analyzed. At both temperatures, the process yields were calculated in two different ways. The yield *Y*_E/G_ is the function of the initial glucose concentration at the beginning of fermentation. The yield *Y*_E/C_ is the function of the initial concentration of biomass in the presaccharification step. According to the *Y*_E/G_ data obtained, it can be confirmed that these values were numerically larger than the theoretical yield of the process (0.51), confirming that the hydrolysis occurred simultaneously with fermentation during the process.

**Table 6:** SSF at 37 and 42 °C with an initial O.D._600 nm_ of 2 in 50 mL of fermentation medium supplemented with 8 % (*w*/*v*) sugarcane bagasse

Yeast strain/ temperature	Initial glucose (g L^−1^)		Final ethanol (g L^−1^)		*Y*_E/C_ (g ethanol/g cellulose)		*Y*_E/G_ (g ethanol/g glucose)	
	37 °C	42 °C	37 °C	42 °C	37 °C	42 °C	37 °C	42 °C
LBM-1	32.56	37.11	22.24	16.63	0.28	0.21	0.68	0.47
CAT-1	33.36	38.99	21.08	22.84	0.26	0.29	0.63	0.59
PE-2	32.93	37.30	19.43	20.31	0.24	0.25	0.59	0.54
UFV-3	36.64	32.60	22.62	16.12	0.28	0.20	0.62	0.51
ATCC 8554	35.38	41.03	22.32	16.95	0.28	0.21	0.63	0.43
CCT 4086	35.00	35.10	21.48	13.30	0.27	0.17	0.61	0.41

SSF tests at both 37 and 42 °C returned very similar *Y*_E/C_ values. However, tests at 37 °C showed a greater production of ethanol than tests at 42 °C. The highest concentration of ethanol at 37 °C was 22.62 g L^−1^ for *K. marxianus* UFV-3, with a yield of 0.28. At 42 °C, the highest concentration of ethanol was 22.84 g L^−1^ for *S. cerevisiae* CAT-1, with a yield of 0.29 (Table 6). Further experiments were performed in bioreactors at 37 °C, with the *S. cerevisiae* CAT-1 strain. The ethanol yield obtained (*Y*_E/C_ = 0.24) in bioreactor was similar to that obtained in Erlenmeyer flasks experiments (*Y*_E/C_ = 0.26).

## Discussion

Tolerance to high temperatures, ethanol and high concentrations of sugars are important features for microorganism producers of ethanol. Thus, the isolation and characterization of yeasts tolerant to these stresses are crucial to the development of processes for ethanol production from lignocellulosic biomass performed simultaneously (SSF) (Ballesteros et al. 2004; Olsson et al. 2006; Sanchez and Cardona 2008). To analyze the effects of temperature and high concentrations of glucose on the growth of yeasts that produce ethanol, experiments were performed under different physiological conditions with *S. cerevisiae* and *K. marxianus* strains. Only *K. marxianus* strains were capable of growing in liquid media at 45 °C (Fig. 2), showing the yeast species’ greater tolerance to higher temperatures compared to *S. cerevisiae* (Abdel-Fattah et al. 2000; Ballesteros et al. 2004; Hong et al. 2007; Nonklang et al. 2008; Singh et al. 1998; Suryawati et al. 2008). At 45 °C, the *S. cerevisiae* strains grew on solid media only at 8 and 16 % (*w*/*v*) glucose concentrations (Table 2). Two events may occur to explain this fact: one is “cross-tolerance”, in which one type of stress confers partial protection against other stresses (Causton et al. 2001; Koedritha et al. 2008; Zakrzewska et al. 2011); the other event is the physical effect of glucose protecting cell membranes from the harmful effects of temperature. This effect of carbohydrates protecting cells against stresses is well documented in the literature for the disaccharide trehalose, but the protective effects of glucose have not yet been described. Thus, additional studies are needed to explain this phenomenon of acquiring temperature tolerance at high glucose concentrations. In assessing the specific growth rates, it was noticed that the *K. marxianus* strains had much higher values compared to *S. cerevisiae* strains (Table 4). *K. marxianus* yeast strains grew similarly to one another. These thermotolerance data were consistent with the literature because other *K. marxianus* strains have been reported to grow rapidly at temperatures above 40 °C (Fonseca et al. 2008; Rocha et al. 2011; Rodrussamee et al. 2011).

When evaluating growth in ethanol, *S. cerevisiae* strains were more tolerant (8 % v/v) compared to *K. marxianus* strains (4 % v/v) (Figs. 3 and 4, respectively). *S. cerevisiae* showed less tolerance to ethanol at temperatures greater than 37 °C (Fig. 3). This effect may be due to the combination of temperature and ethanol stresses. High concentrations of ethanol can damage the cell wall, changing the permeability of the plasma membrane and the various transport systems, such as the glucose transport system. High temperatures can damage the membrane and can cause protein denaturation and aggregation. Together, these factors inhibit growth and diminish the viability of the cultures (Ma and Liu 2010; Singer and Lindquist 1998).

*K. marxianus* and *S. cerevisiae* strains were evaluated for their capacities to ferment sugarcane bagasse via the simultaneous saccharification and fermentation process (Table 6). SSF tests at both 37 °C and 42 °C returned very similar *Y*_E/C_ values. However, tests at 37 °C resulted in greater ethanol production than tests at 42 °C, which could be related to the greater fermentative capacity of strains at the temperature 37 °C. In the presence of glucose, the concentration and yield of ethanol were higher at 37 °C than at 42 °C, with yields close to the theoretical yields.

Upon analyzing the ethanol yields obtained (Table 6), it was noted that the values were similar to values already described in the literature for *S. cerevisiae* and *K. marxianus* isolates (De Souza et al. 2012; Santos et al. 2010). (Faga et al. 2010), using *K. marxianus* strains, obtained an ethanol concentration of approximately 19 g L^−1^ and an ethanol yield of 0.26 at 45 °C using *Panicum virgatum* as substrate. In experiments conducted with the *S. cerevisiae* D_5_A strain, concentration and yield were close to 14 g L^−1^ and 0.19 g g^−1^, respectively. (De Souza et al. 2012), using two of the strains used in this study, *K. marxianus* UFV-3 and *S. cerevisiae* LBM-1, obtained yields of 0.15 g g^−1^ for *S. cerevisiae* LBM-1 at 37 °C and 0.18 g g^−1^ for *K. marxianus* UFV-3 at 42 °C. In this work, the same conditions previously used by (De Souza et al. 2012) were used, but with a single change in the volume of medium to be fermented. The present work was performed with 50 mL fermentation medium, while (De Souza et al. 2012) used a volume of 30 mL. Note that a larger volume of medium in Erlenmeyer flasks of the same volume favored the final yield of the SSF process. This effect may be related to the lower rate of evaporation and oxygen diffusion when using larger volumes of growth medium due to less agitation under these conditions. Comparing the yields obtained by Santos et al. (2010) with the yields obtained in this work, the importance of using thermotolerant yeast for the SSF process is highlighted.

In recent years, thermotolerant yeasts intended for use in SSF processes have been isolated and characterized. Some of these strains were able to ferment at 45 °C. However, higher ethanol yields were obtained when the process was performed between 37 and 42 °C. Above 40 °C, yeast viability decreases considerably, which affects ethanol production (Suryawati et al. 2008).

Among the yeast species known and used in fermentation processes, *K. marxianus* is the only one that is able to grow and ferment sugars at temperatures above 40 °C (Abdel-Fattah et al. 2000; Ballesteros et al. 2004; De Souza et al. 2012; Hong et al. 2007; Nonklang et al. 2008; Singh et al. 1998; Suryawati et al. 2008). Among the advantages of using thermotolerant yeasts, one can cite reduced cooling costs, exemption from refrigeration units, better hydrolysis yields, reduced risks of contamination, and continuous evaporation of the broth under reduced pressure (Hasunuma and Kondo 2012). Moreover, the use of industrial yeasts can facilitate the viability of the SSF process, as their stability in fermentation mash from different crops are known and they have been selected for their capacity to survive in industrial conditions and to compete with wild strains. The industrial *S. cerevisiae* strains CAT-1 and PE-2 were selected from industrial processes and have been screened through a well-directed yeast selection program (Amorim et al. 2011; Basso et al. 2008). *S. cerevisiae* LBM-1 yeast shows thermotolerance, capacity for growth, and fermentation at 42 °C, which are generally not found in yeasts belonging to the *S. cerevisiae* species; this strain shows promising use in industrial processes because it is wild and has not yet experienced selective industrial pressures. Future studies using *S. cerevisiae* LBM-1 shall aim to increase its tolerance to the stresses inherent in the ethanol production process and verify its stability in mash fermentation. Finally, results of this study demonstrated that industrial *S. cerevisiae* strains (CAT-1 and PE-2) have the potential to produce cellulosic ethanol, once they are able to produce similar ethanol yields to those of thermotolerant strains. In addition, the ethanol production from cellulosic biomass by *S. cerevisiae* CAT-1 in bioreactor was similar to that in Erlenmeyer flasks, indicating that this yeast is a good candidate to produce cellulosic ethanol on a large scale. The industrial strains are more tolerant to ethanol and have already been adapted to industrial conditions. Moreover, the study demonstrates that, although *K. marxianus* strains have fermentative capacities similar to *S. cerevisiae* strains, they have low ethanol tolerance, a characteristic that is an important target for enhancing the performance of this yeast in ethanol production.
